# Hidden Injuries in Emergency Care: What the Emergency Room Did Not See Coming

**DOI:** 10.7759/cureus.112813

**Published:** 2026-07-16

**Authors:** Jane Montague, Bryn E Curcio, Emma Cowles, Amin Suchedina, Gary Schwartz

**Affiliations:** 1 Allopathic Medicine, Nova Southeastern University Dr. Kiran C. Patel College of Allopathic Medicine, Fort Lauderdale, USA; 2 Allopathic Medicine, Nova Southeastern University Dr. Kiran C. Patel College of Allopathic Medicine, Davie, USA; 3 Orthopaedic Surgery, Nova Southeastern University Dr. Kiran C. Patel College of Allopathic Medicine, Fort Lauderdale, USA

**Keywords:** delayed diagnosis, emergency department, missed injuries, secondary survey, trauma

## Abstract

Missed injuries in emergent care and trauma settings detrimentally affect patient outcomes, prolonging hospitalization, delaying treatment, and even increasing mortality risk. Despite standardized trauma protocols, injuries can escape detection during the initial evaluation. Our objective in this article is to review clinical factors that may be associated with missed injury rates in patients. This could possibly identify patterns that may help develop future prevention methods and subsequent improvements in emergency care. A targeted literature review of 14 peer-reviewed studies evaluating unrecognized injuries was conducted. Factors analyzed include patient incapacitation measured using the Glasgow Coma Score (GCS), interhospital transfer, patient's sex, physician training level, and location. Our findings indicated that junior physicians accounted for most reported and overlooked injuries compared with intermediate- and senior-physicians, demonstrating that limited clinical experience may adversely affect patient outcomes. Interhospital transfer was also associated with increased rates of undiagnosed injuries, possibly due to disjointed care, poor communication, or documentation inconsistencies. Surprisingly, patient incapacitation was not associated with increased missed injury rates. Upper extremity injuries, especially those involving the hand and wrist, were the most commonly overlooked injuries, followed by lower extremity injuries. The country of medical care did not appear to relate to differences in undiagnosed injury rates. It is the authors’ belief that missed injuries are a persistent challenge in trauma care and seem to relate mostly to the healthcare system rather than patient factors. Stronger interhospital transfer systems, improved physician training, and standardized tertiary physical evaluations could help improve patient outcomes. Future research should include more large-scale studies, as this review included only 14 articles.

## Introduction and background

Introduction

Missed injuries are a significant problem in emergency settings and adversely affect patient outcomes. These are defined as injuries that are not diagnosed during the emergency department evaluation or the initial hospital admission [1]. Missed injuries delay treatment, resulting in prolonged hospitalizations, increased healthcare costs, and morbidity/mortality rates [2]. This review examines clinical and systemic factors that can contribute to missed injuries, with an emphasis on analyzing physician training, hospital transfers, patient characteristics, and health care system variability.

In the evaluation of trauma patients, health care providers follow the guidelines from the Advanced Trauma Life Support (ATLS) by the American College of Surgeons. This guideline consists of a primary survey, where life-threatening injuries are assessed and immediately treated. The secondary survey occurs after the patient has been stabilized and is a head-to-toe approach to identify any major or minor injuries [3]. Unfortunately, many injuries may go undetected during the secondary trauma survey. The rate of missed or delayed diagnoses ranges from 1.4% to 65%, depending on study definitions, patient populations, and follow-up duration [4].

In emergency situations, altered mental status, priority of injury assessment, and evaluation of hemodynamic instability can all contribute to unidentified injuries upon evaluation [1]. Factors associated with overlooked injuries include altered levels of consciousness, hemodynamic instability, multi-trauma presentation, low index of suspicion, inadequate radiology exams, alcohol influence, Glasgow Coma Score (GCS), shock, delayed presentation, and misinterpretation of studies [1,3]. Repeated assessments and studies are necessary to ensure a thorough workup [3]. Certain injuries are more frequently missed, such as those to the upper and lower extremities. This review aims to identify associations behind missed injuries to encourage education and prevention in emergency practice.

Methods

We conducted a targeted literature review of 14 peer-reviewed articles, each analyzing cases of missed injuries in clinical settings. The articles’ publication dates range from 1967 to 2025. These articles assessed data from the United States, the Netherlands, Australia, England, Northern Ireland, and India. Our research focuses on five main factors that may increase the risk of overlooking an injury: the patient’s sex, patient incapacitation, transfer between hospitals, level of physician training, and the country of treatment. These topics were chosen after hypotheses of factors that could influence the rate of missed injuries and article review that encouraged further assessment of these factors.

To identify relevant studies, a structured literature search was performed across databases for reports that assessed missed injuries in different settings. Studies were included based on population size, clearly defined methodology, and whether at least one of the five variables was analyzed in the article. The databases PubMed and GoogleScholar (Mountain View, California) were queried, and searches focused on “missed injuries.” Articles were excluded if they focused on a single injury type, lacked broad trauma context, lacked sufficient patient data, had insufficient sample sizes, or did not clearly define a “missed injury.” The articles chosen focused on and detailed missed injuries in emergency settings while meeting exclusion criteria. This was done to maintain consistency across the review. No formal statistical analysis or meta-analysis was performed because this manuscript is designed as a literature review.

## Review

Clinical factors contributing to missed injuries

Patient Incapacitation

Patient incapacitation was assessed through the GCS [5]. A score of 15 indicates that the patient is fully awake and oriented. It was hypothesized that patients who were intoxicated, unconscious, or had altered mental status would have an increased number of missed injuries compared to those who were not. Outside of the purely clinical context, many systemic factors influence the number and degree of unidentified injuries seen in acute patients. Several key factors include the level of physician training, interhospital transfers, and the country of treatment.

Level of Physician Training

**Figure 1:**
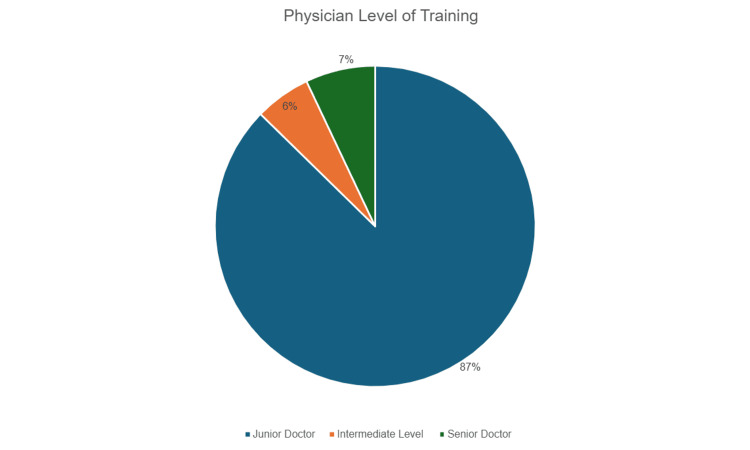
Physician level of training. This figure is based on data from Morrison and Hyland-McGuire and is categorized according to the level of physician training and seniority. The figure was created by the authors using the data from the original publications [6,7].

Trauma patients often sustain multiple injuries simultaneously. Immediate care and urgent diagnostic imaging are necessary. Junior physicians have less experience and may be more susceptible to diagnostic oversight in the presence of more readily identifiable conditions, especially in an acute trauma setting. According to the combined data from Morrison and Hyland-McGuire, 62 missed injuries were attributed to junior physicians, four to intermediate-level physicians, and five to senior doctors [6,7]. These findings are illustrated in Figure 1.

Additionally, subtle findings on diagnostic imaging may require a more trained physician. Injuries such as the posterior shoulder dislocation or locked knee discussed can be missed. Small and complex regions such as the carpal bones may be overlooked [6,8,9,10]. In particular, lunate fractures with a concomitant perilunate injury can lead to an unrecognized diagnosis even when the lunate fracture was present on the original imaging.

Obvious injuries and immediate care require a focused history and physical exam. The senior physician is aware of common pitfalls and missed diagnoses associated with trauma injuries. It was hypothesized that junior physicians are more susceptible to cognitive biases. In the high-acuity environments explored in the literature, it is possible junior physicians became anchored to their initial diagnosis and prematurely closed the diagnostic process [6,7].

Interhospital Transfer

The transfer of patients between healthcare systems is often necessary to properly continue treatment, especially in trauma cases that require specialized resources. However, these transfers are vulnerable to miscommunication, fragmentation of care, and documentation of errors. For severely injured patients requiring interhospital transfer, the rate of undiagnosed injuries can be much higher, with some studies listing it as high as 35% [11].

Handoff miscommunication is a major concern in these instances [11]. Staying within one hospital has the advantage of shared clinical context, but in situations where a transfer is necessary, there can be a risk of misinterpretation. Even if the data and documentation from the original team are preserved, the context can be lost with each successive handoff. Care initiated at one facility must often be transferred and continued at another. This fragmentation of care can lead to confusion of the given plan, reversal of care, delays in treatment, or even a regression in the patient’s current state.

Documentation concerns are also an issue with interhospital transfers. One hospital may have a certain electronic medical record (EMR) that is incompatible or unable to be accessed from the second hospital. There may also be incomplete documentation of the original assessment, which omits critical information and delays care. The healthcare team at the second hospital may assume that the original assessment was comprehensive and try to accelerate treatment. This can lead to inconsistencies in the documentation and care of the patient.

Country of Treatment

The country or environment in which treatment is conducted can influence the number of missed injuries. There are different healthcare systems, infrastructures, and standards of care across regions globally. There is also variability in resources, training, and protocols. When comparing the eight countries discussed in [12], several of these differences can be identified. The journal explores variation in trauma care in Australia, Brazil, Belgium, Canada, the Netherlands, Japan, the United States, and South Africa. The maturity of trauma systems and the extent of registry development are greater in the Netherlands, Canada, and Japan [12]. However, Brazil, Belgium, and South Africa are in earlier stages [12]. Geographical location and layout have bearing on outcomes as well. Canada and Australia have rural populations with greater limitations than other countries [12]. The mechanism of injury can influence findings in the rates of missed injuries. Some countries had higher rates of crime, whereas others had more traffic-related injuries. Disparities in healthcare access were issues highlighted in the United States and South Africa [12]. Major contributors to trauma outcomes were the country’s level of income and access to resources, with low- to middle-income countries making up the majority of trauma-related deaths [12].

A subset of articles investigated trauma care at tertiary rural locations in India [13]. Missed injury rates were 2-3% at the rural centers. This was similar to the 3.4% rate at St George’s Hospital in London [5]. The overlooked injuries in rural trauma centers can be attributed to the lack of specialist care, less advanced imaging modalities, and structure. However, urban major trauma centers will see far more multi-trauma patients in worse conditions, leading to higher rates as well. Resource availability is a primary concern in treatment delivered in rural settings. Inadequate health care supplies and imaging are likely to lead to missed injuries. Improper staffing and less time available per patient heavily influence these findings as well.

Different undiagnosed fracture rates across Emergency Departments in the Netherlands, Taiwan, Norway, the United Kingdom, and the United States have been documented. However, studies looked at different time periods, trauma center types, and resource availability. Although variations between healthcare locations can lead to missed orthopedic injuries, the many confounding variables make a strong comparison challenging.

Patterns of Missed Injuries

**Figure 2:**
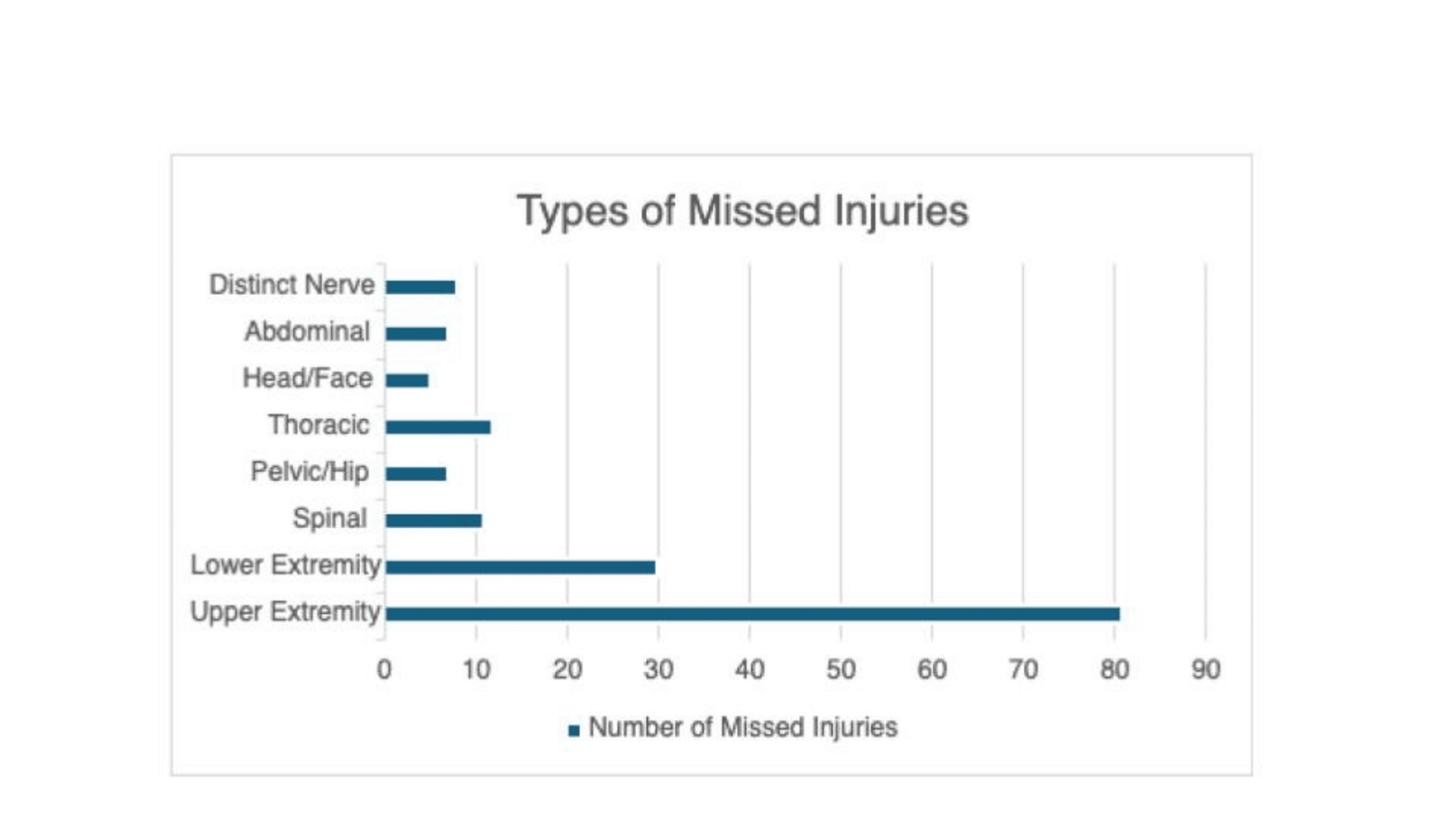
Types of missed injuries. The data shown above were synthesized from all of the studies included in this review examining missed musculoskeletal injuries in emergency departments. The injuries were grouped by anatomic location, and the totals were calculated based on the total number of missed injuries reported for each category. The figure was created by the authors based on aggregated data from references [1-14].

There were 81 upper extremity injuries, 30 lower extremity, 11 spinal, seven pelvic/hip, 12 thoracic, five head/face, seven abdominal, and eight distinct nerve injuries. Of the upper extremity injuries, there were eight shoulders, four elbows, 10 forearms, 12 wrists, 31 hands, and 16 overlapping/miscellaneous injuries. The types of missed injuries are described in Figure 2.

**Figure 3:**
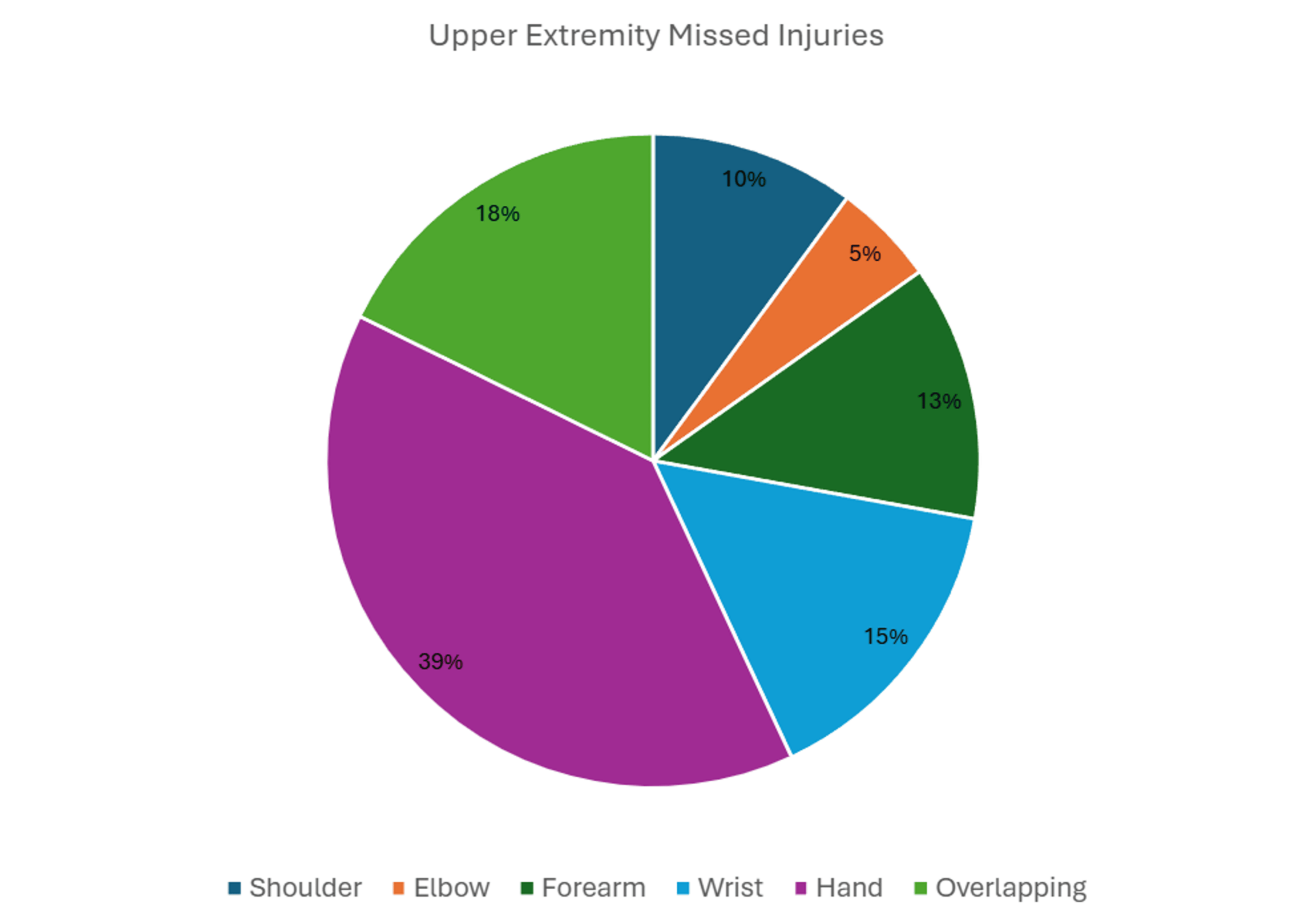
Missed injuries of the upper extremity. The data shown above were synthesized from all of the studies included in this review examining missed upper extremity injuries in emergency department settings. The injuries were grouped by anatomic location (shoulder, elbow, forearm, wrist, hand, and overlapping injuries), and the total percentages were calculated based on the number of missed injuries reported for each category. The figure was created by the authors based on aggregated data from references [1-14].

**Figure 4:**
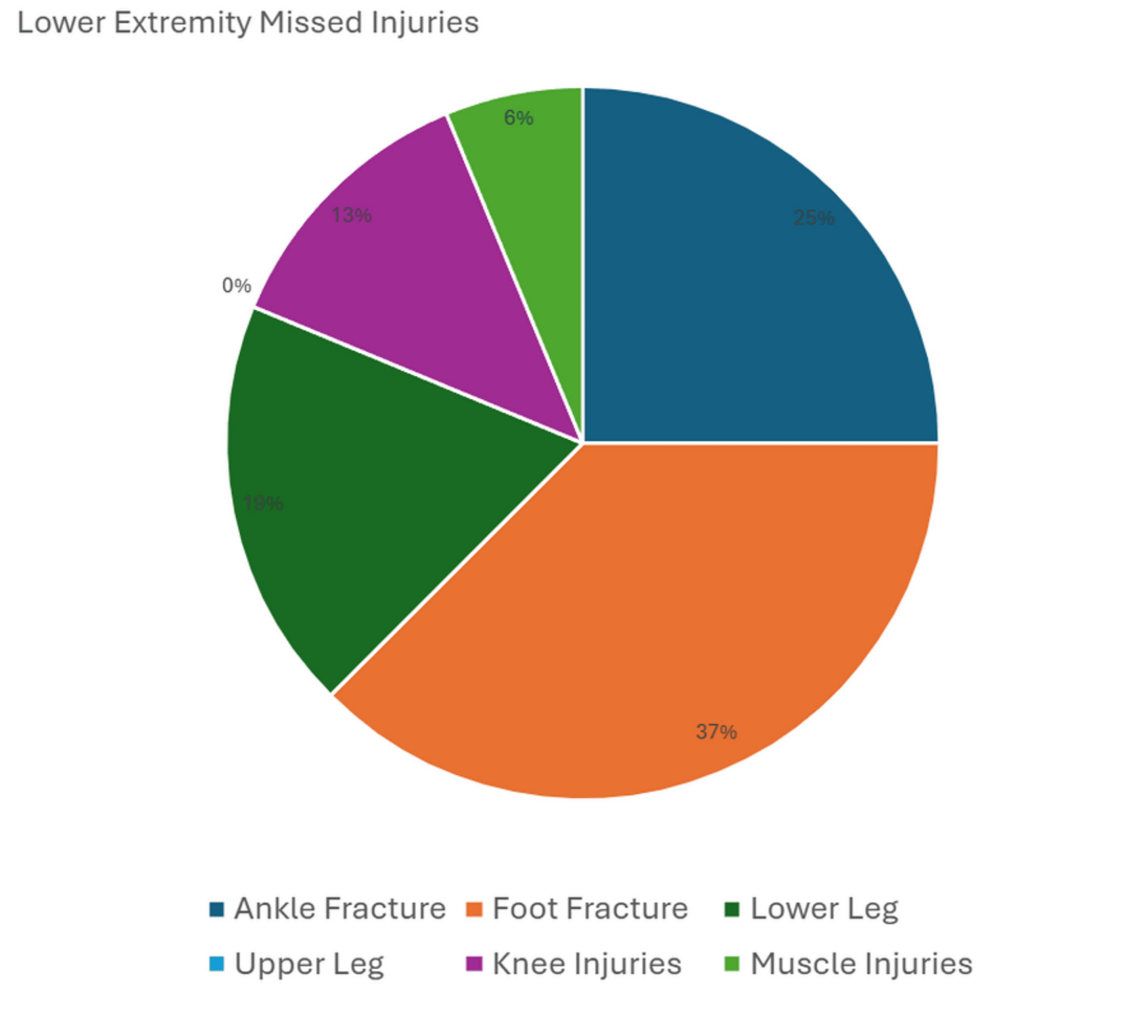
Missed injuries of the lower extremity. The data shown above were synthesized from all of the studies included in this review examining missed lower extremity injuries in emergency department settings. The injuries were grouped by anatomic location and fractures versus muscular injuries (ankles, feet, upper/lower legs, knees, and specific muscle injuries). The total percentages were calculated based on the number of missed injuries reported for each category. The figure was created by the authors based on aggregated data from references [1-14].

After review, we found that upper extremity injuries, particularly those to the hand and wrist, were the most overlooked among all reported cases (Figure 3). This was followed by lower extremity injuries that consisted primarily of injuries to the feet (Figure 4). Upon reflection, this distribution is consistent with the prioritization inherent in trauma evaluation, where more vital regions of the body, including the head and torso, are assessed more thoroughly during the primary survey. As a result, injuries to the extremities may be overlooked, particularly in the presence of life-threatening injuries. Of note, while less common, several cases of injuries to the pelvis, thorax, head/face, abdomen, and distinct nerves were also reported as initially missed.

Clinical implications

We believe these findings indicate the need for standardized protocols for emergency imaging and primary, secondary, and tertiary full-body evaluations of high-acuity trauma patients. Reducing missed injuries in the emergency department starts with maintaining a high index of suspicion and taking a systematic approach to every patient. Even in a busy emergency setting, careful history-taking, thorough physical examinations, and reassessing patients when symptoms change or persist can help identify injuries that are not obvious during the initial evaluation. Current standard trauma imaging protocols often focus on the chest, abdomen, and pelvis, which may contribute to missed distal extremity injuries unless specifically indicated [14]. Imaging protocols could be updated to require imaging of less-acute areas once the patient is stable to prevent diagnostic errors.

Furthermore, these findings underscore the importance of a thorough secondary survey and highlight the role of a tertiary survey in identifying injuries that may not be apparent during the initial evaluation. For example, protocols outlining a focused evaluation of the extremities could be implemented once the patient has been stabilized to guide physicians in re-evaluation methods to prevent significant functional impairments. These protocols could include checklists provided by the hospital or programmed into the EMR system.

Improved handoff communication protocols can also help prevent errors during transfer and ensure all healthcare providers are aware of the extent of the patient’s injuries. This information, if standardized, can guide the accepting medical team on the best next steps for evaluation and care of the patient. Lastly, additional training for junior physicians can enhance their rapid full-body examination skills in a trauma setting, preventing future undiagnosed injuries.

Additionally, physicians can help prevent reinjury by ensuring patients receive appropriate treatment, clear discharge instructions, and timely follow-up care. When appropriate, referral to physical therapy or specialist care can help patients safely return to normal activities while reducing the risk of recurrent injury.

Limitations of current literature

This literature review primarily evaluated 14 studies. A larger number of studies would help identify patterns in missed injuries and lead to improvements. Although these studies represent a variety of geographical regions and patient populations, several studies focus particularly on hand and wrist injuries. Furthermore, two studies represent more classic literature, and several studies were published more than five years ago. More recent data can give a better representation of current missed orthopedic injury rates and any systemic improvements that have been made. Confounding variables are not controlled, particularly when comparing rates between sexes and geographical regions. Potential bias can be identified in studies that collect data from their own patient populations. Reporting missed injuries and lapses in care may reflect poorly on the institutions involved, so a degree of understated findings may be present. The results, data, and charts made from the articles used are not meant as a statistical comparison but rather a descriptive review of the injuries documented in reviewed articles.

Future directions

While there is a growing body of literature regarding missed injuries in the emergency department, there remain significant gaps. To begin, large multicenter studies are needed to better understand diverse patient populations and enhance the generalizability of findings. Additionally, establishing a standardized definition of a “missed injury” would improve comparability across studies and allow for more accurate reporting of incidence rates. There is also a growing interest in the emerging role of artificial intelligence (AI) technologies in reducing diagnostic error. AI-assisted imaging devices, for example, may help identify subtle or overlooked injuries, particularly in high-volume emergency settings. Further investigation is needed to evaluate the efficacy and safety of this approach, as well as the integration of AI into clinical decision-making.

System-level improvements also remain a key area of focus. Namely, optimizing handoff communication, particularly during hospital transfers, may reduce information loss and improve continuity of care. Additionally, structured protocols and standardized documentation systems may help reduce missed injuries. Finally, training in undergraduate and graduate medical education should include simulation-based learning that prioritizes standardized trauma surveys as a means of minimizing diagnostic inaccuracy and cognitive bias.

## Conclusions

Missed injuries remain a challenge in emergency and trauma care, with significant implications for patients, providers, and healthcare systems. This review highlights that systemic factors play a stronger role in the occurrence of missed injuries than patient factors. Specifically, the level of physician training and interhospital transfers consistently contribute more to overlooked injuries than patient sex and incapacitation. These findings suggest that undiagnosed injuries are not solely the result of individual oversight but reflect health care system-level vulnerabilities.

Addressing this issue requires a multifaceted approach that includes stronger clinical training, improved communication during transferring of care, and reinforcing adherence to standardized trauma protocols. Continued efforts toward system-wide improvements and evidence-based interventions are important in reducing the incidence of missed injuries and, ultimately, improving patient care and outcomes.
